# Oxidative Stress Accumulates in Adipose Tissue during Aging and Inhibits Adipogenesis

**DOI:** 10.1371/journal.pone.0018532

**Published:** 2011-04-14

**Authors:** Hannes M. Findeisen, Kevin J. Pearson, Florence Gizard, Yue Zhao, Hua Qing, Karrie L. Jones, Dianne Cohn, Elizabeth B. Heywood, Rafael de Cabo, Dennis Bruemmer

**Affiliations:** 1 Saha Cardiovascular Research Center, University of Kentucky College of Medicine, Lexington, Kentucky, United States of America; 2 Graduate Center for Nutritional Sciences, University of Kentucky College of Medicine, Lexington, Kentucky, United States of America; 3 Laboratory of Experimental Gerontology, National Institute on Aging, National Institutes of Health, Baltimore, Maryland, United States of America; Pennington Biomedical Research Center, United States of America

## Abstract

Aging constitutes a major independent risk factor for the development of type 2 diabetes and is accompanied by insulin resistance and adipose tissue dysfunction. One of the most important factors implicitly linked to aging and age-related chronic diseases is the accumulation of oxidative stress. However, the effect of increased oxidative stress on adipose tissue biology remains elusive. In this study, we demonstrate that aging in mice results in a loss of fat mass and the accumulation of oxidative stress in adipose tissue. *In vitro*, increased oxidative stress through glutathione depletion inhibits preadipocyte differentiation. This inhibition of adipogenesis is at least in part the result of reduced cell proliferation and an inhibition of G_1_→S-phase transition during the initial mitotic clonal expansion of the adipocyte differentiation process. While phosphorylation of the retinoblastoma protein (Rb) by cyclin/cdk complexes remains unaffected, oxidative stress decreases the expression of S-phase genes downstream of Rb. This silencing of S phase gene expression by increased oxidative stress is mediated through a transcriptional mechanism involving the inhibition of E2F recruitment and transactivation of its target promoters. Collectively, these data demonstrate a previously unrecognized role of oxidative stress in the regulation of adipogenesis which may contribute to age-associated adipose tissue dysfunction.

## Introduction

The prevalence of type 2 diabetes is increasing annually, which is thought to be due to physical inactivity, obesity, population growth, and aging [1], [2]. In particular the increased prevalence of obesity has been recognized as a major risk factor for type 2 diabetes [3]. However, according to recent estimates, the number of patients with type 2 diabetes will more than double by 2030, even if the prevalence of obesity remains constant [4]. Currently more than half of the 20 million U.S. adults with type 2 diabetes are above age 60, and the largest increase in type 2 diabetes prevalence is expected in the elderly [1], [5], [6]. Considering this evidence, it has been estimated that by 2050 there will be an additional 18 million people with type 2 diabetes in the U.S. alone as a result of increased longevity [7].

While the mechanisms underlying the relationship between obesity and type 2 diabetes are beginning to emerge [8], [9], the link between aging and type 2 diabetes remains elusive. A decline in glucose tolerance as part of human aging was first noted in 1921 [10], and diminished insulin sensitivity is now recognized as a primary cause of the age-related impairment in glucose metabolism [11], [12]. One of the hallmarks of both physiological aging and genetic forms of premature aging is the loss of insulin sensitive subcutaneous adipose tissue [13], [14]. This age-related decline in subcutaneous fat depot size is thought to be due to altered replication and differentiation of preadipocytes [15], [16], [17], [18]. The consequence of this adipose tissue dysfunction is the redistribution of fat from subcutaneous depots not only to intra-abdominal visceral depots but also to ectopic sites including muscle and liver [19], [20], [21]. Since impaired adipogenesis and ectopic lipid accumulation are closely related to insulin resistance [22], [23], a deterioration of adipose tissue function with aging is likely to contribute to impaired glucose homeostasis. However, the mechanisms governing age-related adipose tissue dysfunction and the limited regenerative capacity of adipose tissue remain to be elucidated.

One of the key hypotheses postulated as the cause for both organismal aging and age-related chronic diseases is the accumulation of oxidative stress [24]. Reactive oxygen species (ROS) accumulating over a lifetime can inflict direct cellular damage and influence various signaling pathways and transcriptional programs regulating key development processes including proliferation, differentiation, senescence, and apoptosis [25]. In addition to the accumulation of oxidative damage during a life span, aging is directly associated with an impaired antioxidant defense mechanism. For example, levels of glutathione (GSH), one of the most important cellular antioxidant defense mechanisms, decline during aging in humans and mice, accompanied by various age-related pathologies [26], [27], [28], [29]. Although increased ROS production has been documented in adipose tissue of premature aging syndromes [30] and is associated with altered glucose homeostasis [31], it remains unknown whether oxidative stress accumulates during physiologic aging and alters adipose tissue biology.

In the present study, we demonstrate that aging in mice results in a loss of adipose tissue mass and the accumulation of oxidative stress. We further establish that increased oxidative stress inhibits the differentiation of preadipocytes by preventing their mitotic clonal expansion and entry into S-phase of the cell cycle. The molecular mechanism underlying this inhibition of cell cycle progression by oxidative stress involves a transcriptional repression of E2F target genes.

## Materials and Methods

### Ethics statement

All procedures were performed in accordance with approved institutional protocols and were approved by the Institutional Animal Care and Use Committee of the National Institute on Aging (277-LEG-2010) and the University of Kentucky (00767M2004).

### Cell culture

3T3-L1 preadipocytes (ZenBio) were maintained in DMEM supplemented with 10% newborn calf serum. Cell differentiation was induced using a standard protocol [32]. Briefly, two-day post confluent cells were treated with 1 µM dexamethasone, 0.5 mM Isobutylmethylxanthine, 10 µg/ml insulin and 10% fetal bovine serum (FBS) for two days, followed by treatment with 10% FBS and insulin for two additional days. Finally, the medium supplemented with 10% FBS was renewed every other day. *L*-buthionine-(*S,R*)-sulfoximine (BSO, Sigma) was dissolved in diH2O and filtered before use in cell culture. Cells were treated with BSO after reaching confluence to day 2 or day 7 of differentiation, as indicated.

### Isolation and differentiation of stromal vascular cells from subcutaneous adipose tissue

Subcutaneous inguinal white adipose tissue was isolated from eight week old C57BL/6 mice, pooled, and minced in HEPES-buffered DMEM (Invitrogen) supplemented with 10 mg/ml fatty acid poor BSA (Sigma). Samples were incubated for 60 min with 0.03 mg/ml Liberase 3 (Roche) at 37°C on an orbital shaker. The solution was passed through a sterile 100 µm nylon mesh and centrifuged at 500 g for 10 min. The cell pellet was resuspended in 5 ml erythrocyte lysis buffer, incubated for 5 min at room temperature, and centrifuged at 500 g for 5 min. Cells were resuspended in DMEM with 10% fetal bovine serum, counted, and plated on 12-well plates at a density of 1×10^5^ cells/well. Two days after reaching confluence, cells were differentiated using adipocyte basal medium (ZenBio) and a standard differentiation protocol.

### Detection of oxidative stress

Twenty four hours after initiation of differentiation cells were washed twice with PBS and incubated with 10 µM 2′,7′-dichlorofluorescin diacetate (H_2_DCFDA) for 30 min at 37°C. Cells were washed again with PBS and collected for FACS analysis. Cells were analyzed at excitation/emission wavelengths of 488/525 nm using a FACSCalibur sorting system (Becton Dickinson).

### GSH/GSSG-assay

GSH/GSSG-ratio was analyzed in epididymal adipose tissue using the Bioxytech GSH/GSSG-412 kit (Oxis Research). Tissue was homogenized by sonication with or without the thiol-scavenging reagent 1-methyl-2-vinylpyridinium trifluoromethanesulfonate (M2VP). Samples were deproteinized with an equal volume of 10% metaphosphoric acid, centrifuged, and the supernatant was analyzed for GSH and GSSG concentrations using a spectrophotometer at 412 nm over 3 min. Concentrations were calculated by comparison to GSSG standards and normalized to protein content. GSH/GSSG-ratio was calculated using the following formula: (total GSH – 2GSSG)/GSSG.

### Oil red O staining

On day 7 of differentiation cells were washed with PBS and fixed for 10 min in 10% formalin. Oil-red-O (0.5% in isopropanol) was diluted with water (3∶2), filtered, and incubated with fixed cells for 2 h at room temperature. Cells were washed, and Oil-red-O was extracted with isopropanol for quantification using a spectrophotometer at 510 nm.

### Cell cycle analysis

Twenty four hours after initiation of differentiation cells were fixed for 30 min in ice cold 70% ethanol. Cells were centrifuged and resuspended in PBS supplemented with 40 µg/ml RNase. After incubation for 30 min at 37°C cells were centrifuged again and resuspended in PBS containing 50 µg/ml propidium iodide. Cell cycle distribution was analyzed by FACS using a FACSCalibur sorting system (Becton Dickinson).

### Cell proliferation assays

To measure the proliferative capacity of preadipocytes during the mitotic clonal expansion cells were counted at day 0 and day 3 of the differentiation process using a hemocytometer. In addition, proliferation was assessed by analyzing cell division using 5(6)-carboxyfluorescein diacetate *N*-succinimidyl ester (CFSE). Two day confluent cells were stained with 0.5 µM/l CFSE for 10 min at 37°C and washed in PBS. One cell plate was immediately fixed in 10% formalin and stored in 80% ethanol until FACS analysis to determine the baseline fluorescence. The remaining cells were subjected to the differentiation protocol and analyzed for CFSE content after three days by FACS [33].

### RNA isolation and quantitative real-time RT-PCR

RNA was isolated and reverse transcribed as described [34]. Quantitative real-time PCR analysis of target gene expression was performed using an iCycler and SYBR Green I system (Bio-Rad) as described [34]. Each sample was analyzed in triplicate and normalized to mRNA expression of the house-keeping gene TFIIB. The following primer sequences were used: MCM7 (forward: 5′-TGTGGGGCAGAGACCTAC-3′ and reverse: 5′-CTGGGCAATCCTTGTGTT-3′), cyclin A2 (forward: 5′-CGCAGCAGAAGCTCAAGAC-3′ and reverse: 5′-CTTGCTGCGGGTAAAGAGAC-3′), aP-2 (forward: 5′-GGCCAAGCCCAACATGATC-3′ and reverse: 5′-CACGCCCAGTTTGAAGGAAA-3′) and TFIIB (forward: 5′ -CTCTCCCAAGAGTCACATGTCC-3′ and reverse: 5′-CAATAACTCGGTCCCCTACAAC-3′).

### Western blotting

Western Blotting was performed as described previously [35] using the following antibodies: p21 ab7960 (Abcam), p27^Kip1^ ab7961 (Abcam), MCM7 sc-9966 (Santa Cruz), CyclinA ab38 (Abcam), GAPDH sc-25778 (Santa Cruz), Phospho-Rb (Ser807/811) 9308S (Cell Signaling), E2F1 sc-193 (Santa Cruz).

### Transient transfections

3T3-L1 preadipocytes were seeded in 6-well plates and transfected two days after reaching confluence. Transient transfections were performed using Lipofectamine 2000 (Invitrogen) and 1.5 µg DNA of a luciferase reporter plasmid driven by the full length MCM7 promoter [36]. Transfection efficiency was normalized to renilla luciferase activities generated by cotransfection with 1.5 ng/well pRL-CMV (Promega). After 4 h the medium was changed to standard differentiation medium. Luciferase activity was assayed 24 h after initiation of differentiation using a Dual Luciferase Reporter Assay System (Promega).

### Chromatin immunoprecipitation assays

Chromatin immunoprecipitation (ChIP) assays were performed using the Magnify-ChIP-System (Invitrogen). Two day confluent stromal vascular cells were stimulated with standard differentiation medium. Cells were harvested at the indicated time points and soluble chromatin was prepared. Chromatin was immunoprecipitated using an antibody (5 µg) directed against E2F-1 (sc-193X, Santa Cruz) or control IgG (provided in the ChIP-kit). Final DNA extractions were PCR-amplified using the following primer pairs that cover the E2F-1 consensus site in the MCM7-promoter: forward: 5′-CGCTTTAAGAAACACTCCTCCCACAC-3′ and reverse: 5′-GCCAGCCCCTAACTTTAACCAATCAATG-3′.

### Aging cohort

Male C57BL/6 mice were obtained from The Jackson Laboratory at 6 weeks of age and allowed to age normally at the National Institute on Aging in Baltimore, MD according to approved animal protocols and NIH guidelines. The mice were housed in an environmentally-controlled vivarium maintained between 68–72°F with unlimited access to food (Diet 2018; Harlan Teklad) and water under a controlled photoperiod (12 hr light; 12 hr dark).

### Body composition

Measurements of fat mass in C57BL/6 mice aged 2.5–29 months were acquired as part of a cross-sectional study by nuclear magnetic resonance using the Minispec LF90 (Bruker Optics).

### Statistics

ANOVAs using one-way or two-way ANOVA with Bonferroni's *t* test for post hoc analysis and paired or unpaired *t* test were performed for statistical analysis as appropriate. Data were reported as means ± SEM. *P* values <0.05 were considered statistically significant.

## Results

### Aging in mice results in a loss of fat mass and increased oxidative stress in adipose tissue

**Figure 1:**
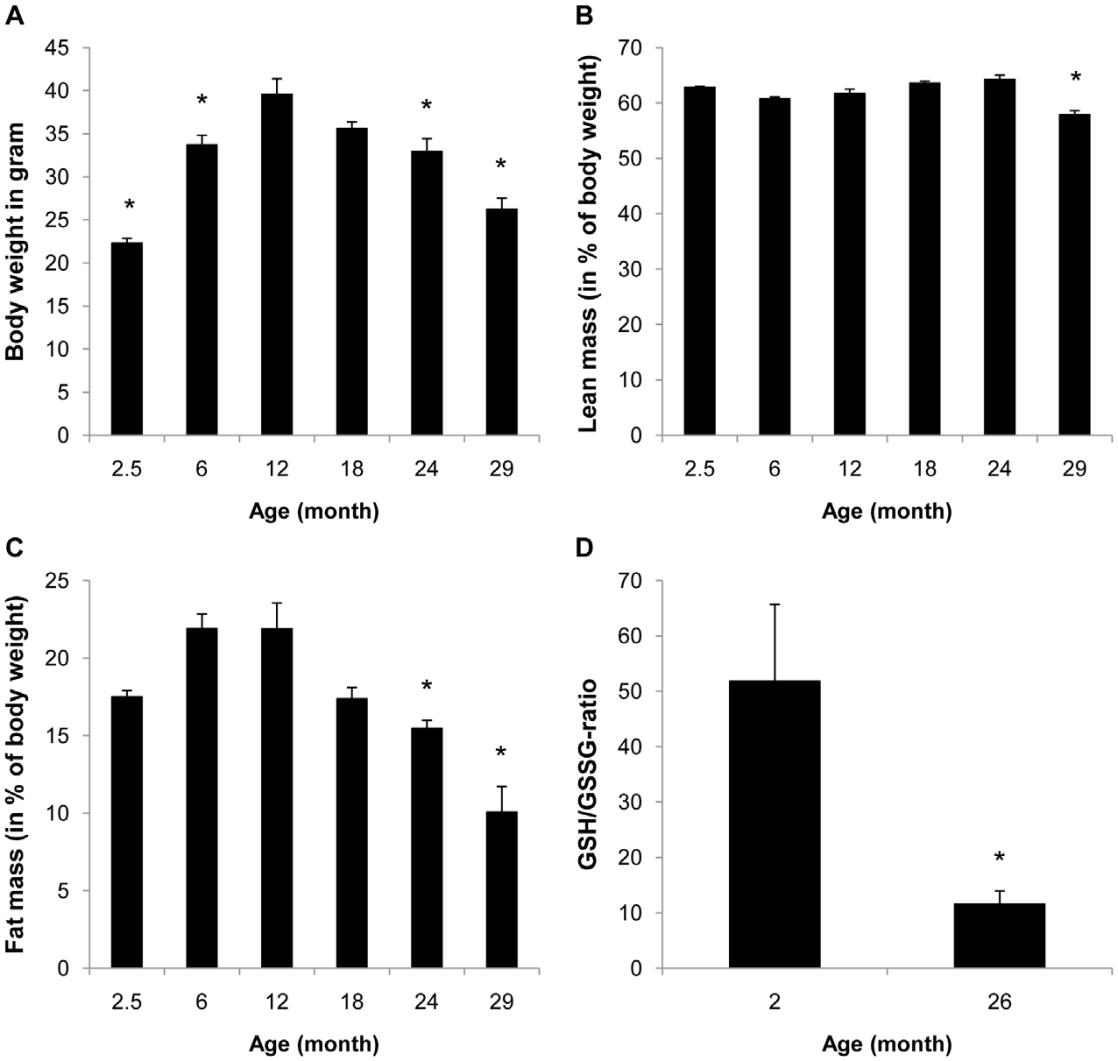
Aging is associated with decreased fat mass and increased oxidative stress in mice. A-C: Body weight and body composition were analyzed in C57BL/6 mice fed a regular chow diet at the indicated age (n = 10 for each age group except n = 9 and 8 for 24 and 29 month, respectively, due to differences in longevity). D: GSH/GSSG-ratio was measured in epididymal adipose tissue of 2 month (n = 7) and 26-month old (n = 5) mice. All results are presented as mean ± SEM (* p<0.05 vs. 12 month in A–C or 2 month in D).

To determine whether aging is associated with changes in body mass and composition, we first followed weight and fat mass in aging C57BL/6 mice. As depicted in Figure 1A, after an initial increase in body weight during the first year there was a continuous decline in body mass in the second and third year of life. Analysis of body composition revealed that this decline in body mass was primarily due to a loss of fat mass (Figure 1B–C). At 29 months of age, the fat mass of C57BL/6 mice was reduced to approximately 30% of the peak fat mass at 12 months of age.

Since oxidative damage constitutes one of the most studied hallmarks of aging and is implicitly linked with age-related disease, we next analyzed whether aging results in increased oxidative stress in adipose tissue. As shown in Figure 1D, the ratio of reduced to oxidized glutathione in adipose tissue decreases significantly in 26-month old mice, representing a shift to increased oxidation.

### Oxidative stress inhibits adipogenesis in 3T3-L1 preadipocytes and stromal vascular cells

**Figure 2:**
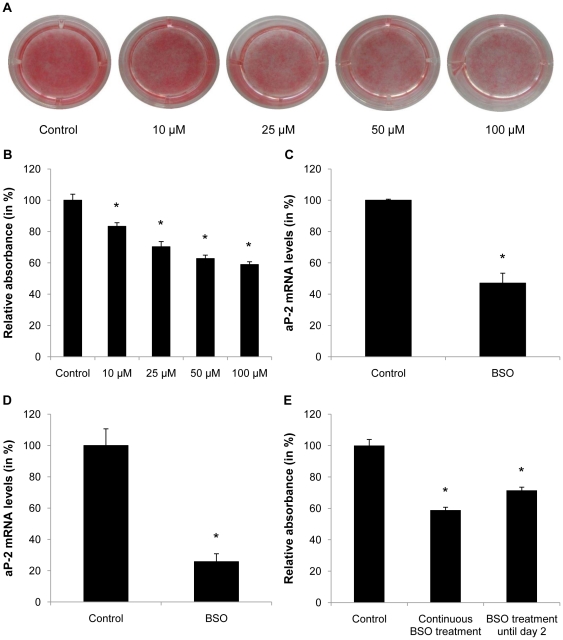
BSO treatment inhibits adipogenesis. A and B: Confluent 3T3-L1 cells were treated with different doses of BSO for 2 days and induced to differentiate. BSO treatment was continued until day 7 of differentiation. Differentiated cells were stained with Oil-red-O and absorbance was measured at 510 nm using a spectrophotometer. C: Confluent 3T3-L1 cells were treated with 100 µM BSO for 2 days and induced to differentiate. BSO treatment was continued until day 7 of differentiation. On day 7 mRNA was harvested and aP-2 expression was analyzed by real-time RT-PCR. D: Confluent subcutaneous stromal vascular cells were treated with 10 µM BSO. After 2 days cells were induced to differentiate and treated with BSO until day 7 of differentiation. On day 7 mRNA was harvested and aP-2 expression was analyzed. E: 3T3-L1 cells were treated with 100 µM BSO for 2 days and induced to differentiate. BSO treatment was continued until day 2 or day 7 of differentiation as indicated. On day 7 cells were stained with Oil-red-O and absorbance was measured. All results are presented as mean ± SEM (* p<0.05 vs. control).

Based on the observation that aging is associated with a decline in fat mass and a concomitant increase in oxidative stress, we next analyzed whether oxidative stress affects adipogenesis. The GSH-dependent ROS scavenging network represents one of the key host defense mechanisms against oxidative stress [27]. Oxidative stress in conjunction with GSH depletion is associated with various diseases and can be achieved pharmacologically to increase endogenously produced oxidative stress using BSO, a specific and irreversible inhibitor of glutamate cysteine ligase [37]. As depicted in Fig. 2A and B, glutathione depletion using BSO significantly inhibited the differentiation of post-confluent 3T3-L1 preadipocytes. Correspondingly, BSO treatment reduced mRNA expression of the adipocyte marker aP-2 after 7 days of differentiation in 3T3-L1 preadipocytes (Figure 2C). Similar results were observed in murine preadipocytes derived from the stromal vascular fraction of inguinal (Figure 2D) as well as epididymal fat pads (data not shown) indicating that the observed inhibition of adipocyte differentiation is also applicable to primary preadipocytes. Due to their significantly higher differentiation capacity *in vitro*, further experiments in primary preadipocytes were performed in SVC of subcutaneous depots. Quantification of Oil-red-O-staining further revealed a comparable inhibition of adipocyte differentiation in 3T3-L1 preadipocytes exposed to BSO treatment either continuously until day 7 or only until day 2 of differentiation (Figure 2E). These results indicate that the inhibition of adipogenesis by BSO occurs at least in part during the initial phase of differentiation, which represents the mitotic clonal expansion phase required for preadipocyte differentiation.

### Glutathione depletion induces oxidative stress

**Figure 3:**
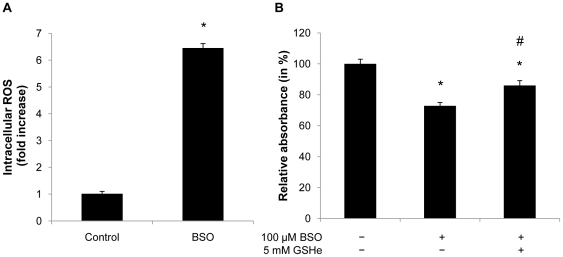
Glutathione depletion induces intracellular ROS formation during differentiation. A: Confluent 3T3-L1 cells were treated with 100 µM BSO. After 2 days BSO treatment was renewed and cells were induced to differentiate. 24 h after hormonal induction cells were incubated with 10 µM H_2_DCFDA and analyzed by FACS. B: Confluent 3T3-L1 cells were treated with 100 µM BSO until day 2 of differentiation. 5 mM GSH-ester was added one day prior to differentiation. On day 7 of differentiation cells were stained with Oil-red-O and analyzed using a spectrophotometer. All results are presented as mean ± SEM (* p<0.05 vs. control, # p<0.05 vs. BSO).

To document specificity of the BSO compound with respect to increased oxidative stress and glutathione depletion, we next measured intracellular ROS levels in 3T3-L1 adipocytes using H_2_DCF-DA. As expected, BSO treatment increased ROS levels by 6-fold 24 h after the induction of differentiation (Figure 3A). Furthermore, reconstitution of glutathione levels in BSO-treated 3T3-L1 cells by providing glutathione-ethylester was sufficient to improve the adipogenic capacity (Figure 3B). These data confirm specificity of BSO treatment and demonstrate that glutathione depletion increases oxidative stress in adipocytes and inhibits adipogenesis.

### Oxidative stress inhibits the mitotic clonal expansion

**Figure 4:**
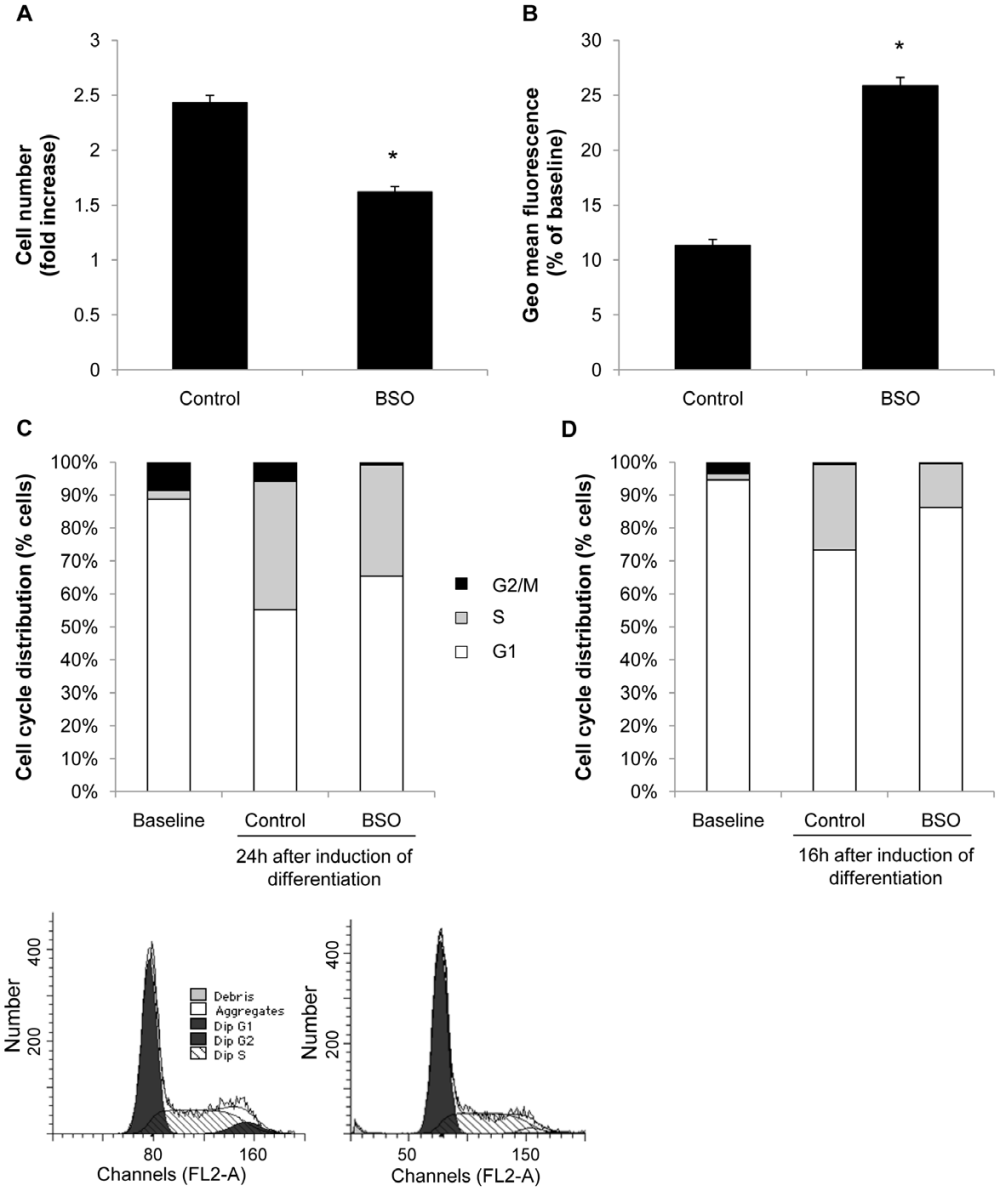
BSO treatment inhibits mitotic clonal expansion. A: Confluent 3T3-L1 cells were treated with 100 µM BSO. After 2 days cells were induced to differentiate. BSO treatment was renewed with every change of medium. Cells were counted at induction and at day 3 of differentiation using a hemocytometer. Cell number did not differ at induction of differentiation. B: Confluent cells were treated with 100 µM BSO for two days. Cells were labeled with CFSE and induced to differentiate. BSO treatment was renewed with every change of medium. On day 3 of differentiation cells were collected and analyzed using FACS. CFSE mean fluorescence declines with cell division, and geometric mean fluorescence is inversely proportional to the proliferation rate. C: Cells were treated as described in A. 24 h after induction of differentiation cell cycle distribution was assessed by DNA staining with propidium iodide and subsequent FACS analysis. Cell cycle distribution did not differ at induction of differentiation (baseline). D: Confluent subcutaneous stromal vascular cells were treated with 10 µM BSO. After 2 days, BSO treatment was renewed and cells were induced to differentiate. Cell cycle distribution was analyzed after 16 h. All results are presented as mean ± SEM (* p<0.05 vs. control).

The observation that oxidative stress inhibits adipocyte differentiation during the first two days of the differentiation process pointed to an inhibition of the mitotic clonal expansion, a prerequisite for adipocyte differentiation. Consistent with this notion, the mitotic expansion of 3T3-L1 preadipocytes following induction of differentiation was significantly decreased by BSO treatment (Figure 4A). To further corroborate that BSO inhibits mitosis of preadipocytes we followed proliferation rates using CFSE labeling, a dye that is inherited equally by daughter cells after division, resulting in the sequential halving of fluorescence with each generation. As depicted in Figure 4B, BSO treated cells exhibited considerably reduced cell division rates evidenced by an increased retention of fluorescence. FACS analysis further revealed that oxidative stress inhibited S phase entry during the mitotic clonal expansion and resulted in an increased number of cells arrested in the G_0_/G_1_ phase (Figure 4C). Similarly, BSO treatment of stromal vascular cells inhibited cell cycle progression (Figure 4D). In concert, these data indicate that oxidative stress inhibits adipocyte differentiation by blocking the mitotic clonal expansion and cell cycle progression.

### BSO treatment results in down-regulation of E2F target genes during mitotic clonal expansion

**Figure 5:**
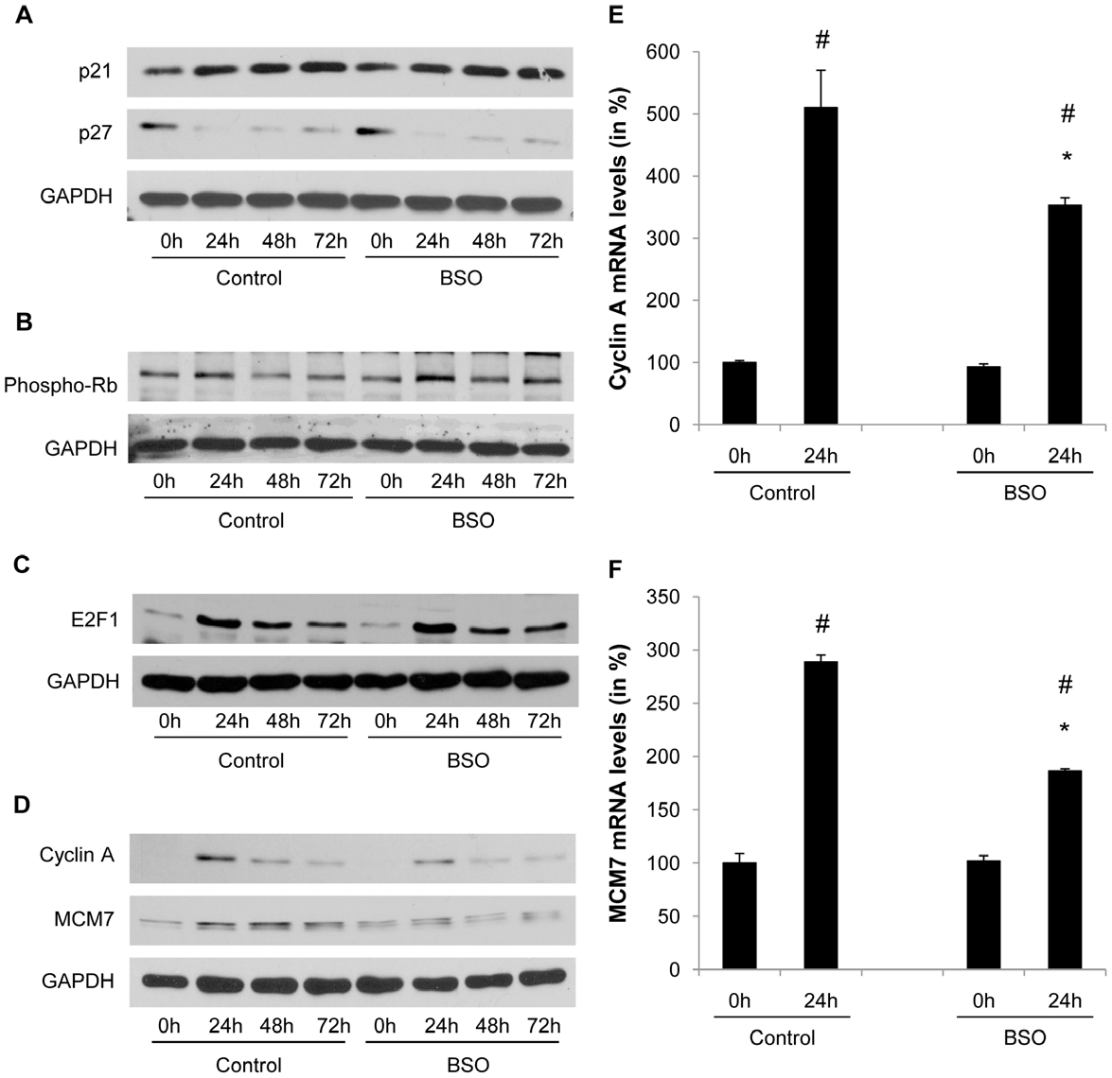
BSO treatment decreases E2F target gene expression. A to D: Confluent 3T3-L1 cells were treated with 100 µM BSO. After 2 days cells were induced to differentiate. BSO treatment was renewed with every change of medium. Whole cell lysate was collected at the indicated time points and analyzed for protein expression of p21, p27, phosphorylated Rb, E2F1, cyclin A, MCM7, and GAPDH. E and F: 3T3-L1 cells were treated as described in A. mRNA was harvested at induction of differentiation and at day 1 of differentiation. MCM7 and cyclin A2 expression was analyzed by real-time RT-PCR. All results are presented as mean ± SEM (* p<0.05 vs. control, # p<0.05 vs. baseline).

G_1_→S phase transition requires phosphorylation of the retinoblastoma protein (Rb) resulting in the transactivation of S phase target genes by the transcription factor E2F [38]. Rb phosphorylation is induced by the formation of cyclin and cyclin-dependent kinase (CDK) complexes and repressed by association with negative regulatory subunits, the CDK inhibitors (CDKI) [39]. As shown in Figure 5A, mitotic clonal expansion during differentiation was associated with a decline in p27^Kip1^ protein levels, and neither the degradation of p27^Kip1^ nor p21 protein levels were affected by increased oxidative stress. During the initial phase of mitotic clonal expression the phosphorylation of Rb was induced; however, this phosphorylation step was not modulated by BSO (Figure 5B). Furthermore, BSO did not alter the induction of E2F1 expression during mitotic clonal expansion (Figure 5C). In contrast, the induction of the downstream S phase E2F target genes cyclin A and MCM7 was considerably reduced at both protein and mRNA expression levels in preadipocytes treated with BSO (Figure 5D–F).

### Oxidative stress alters the E2F-dependent transactivation of the MCM7 target promoter

**Figure 6:**
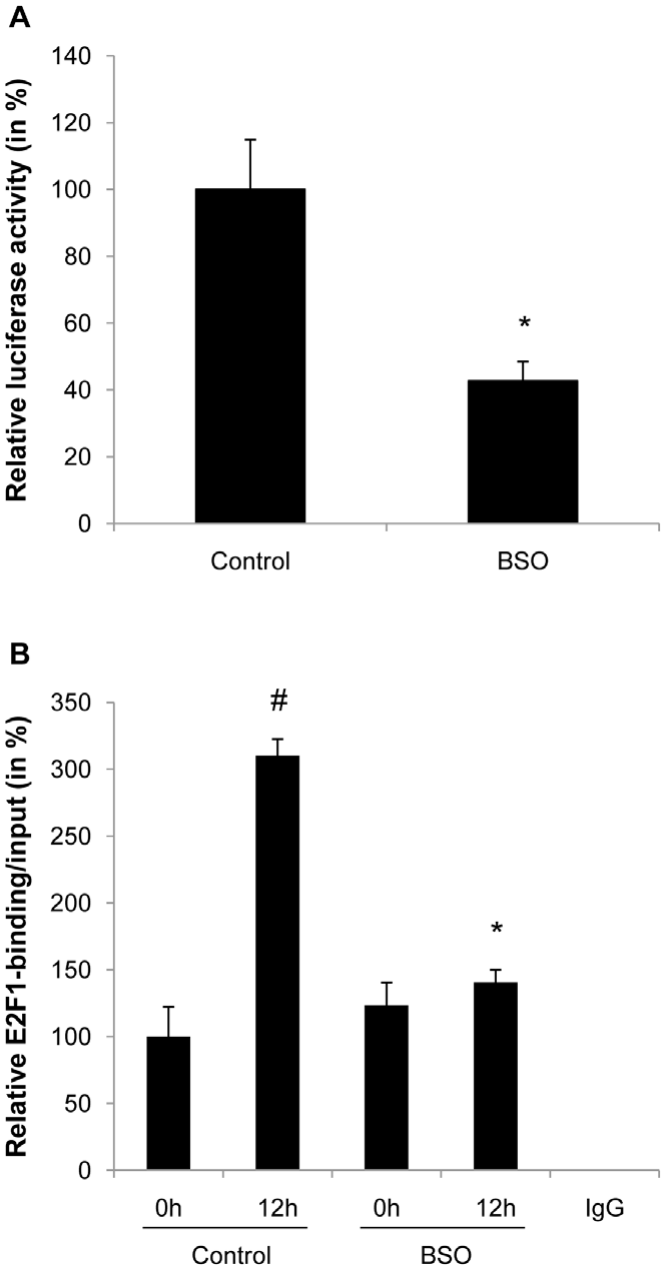
Oxidative stress alters E2F-dependent transactivation of the MCM7 promoter. A: 3T3-L1 cells were treated with 100 µM BSO. After 2 days cells were transfected with a MCM7 promoter luciferase reporter plasmid. Following transfection, cells were induced to differentiate and BSO treatment was renewed. Luciferase activity was assayed 24 h after initiation of differentiation. Transfection efficiency was adjusted by normalizing firefly luciferase activities to renilla luciferase activities generated by cotransfection with pRL-CMV. B: Confluent subcutaneous stromal vascular cells were treated with 10 µM BSO. After 2 days BSO treatment was renewed and cells induced to differentiate. Cells were collected at indicated time points for ChIP assays. After chromatin immunoprecipitation with an E2F antibody or control IgG, quantitative real-time RT-PCR analysis was performed with primer pairs covering the E2F-binding site in the MCM7 promoter. All results are presented as mean ± SEM (* p<0.05 vs. control, # p<0.05 vs. baseline).

To further determine the mechanisms underlying the altered transcription of the bona fide E2F target genes cyclin A and MCM7 by oxidative stress, we next analyzed the promoter activity of these two genes. Exemplified for the MCM7 promoter in Figure 6A, BSO treatment profoundly decreased the activity of a luciferase reporter driven by the MCM7 promoter. Furthermore, ChIP assays demonstrated that oxidative stress altered the induction of E2F binding to its consensus site in the MCM7 promoter during the early differentiation process (Figure 6B). Collectively, these data indicate that the inhibition of cell cycle transition by oxidative stress is at least in part the result of an altered *trans*activation and transcriptional silencing of E2F target genes.

### Aged adipose tissue displays reduced expression of E2F target genes and adipocyte differentiation markers

**Figure 7:**
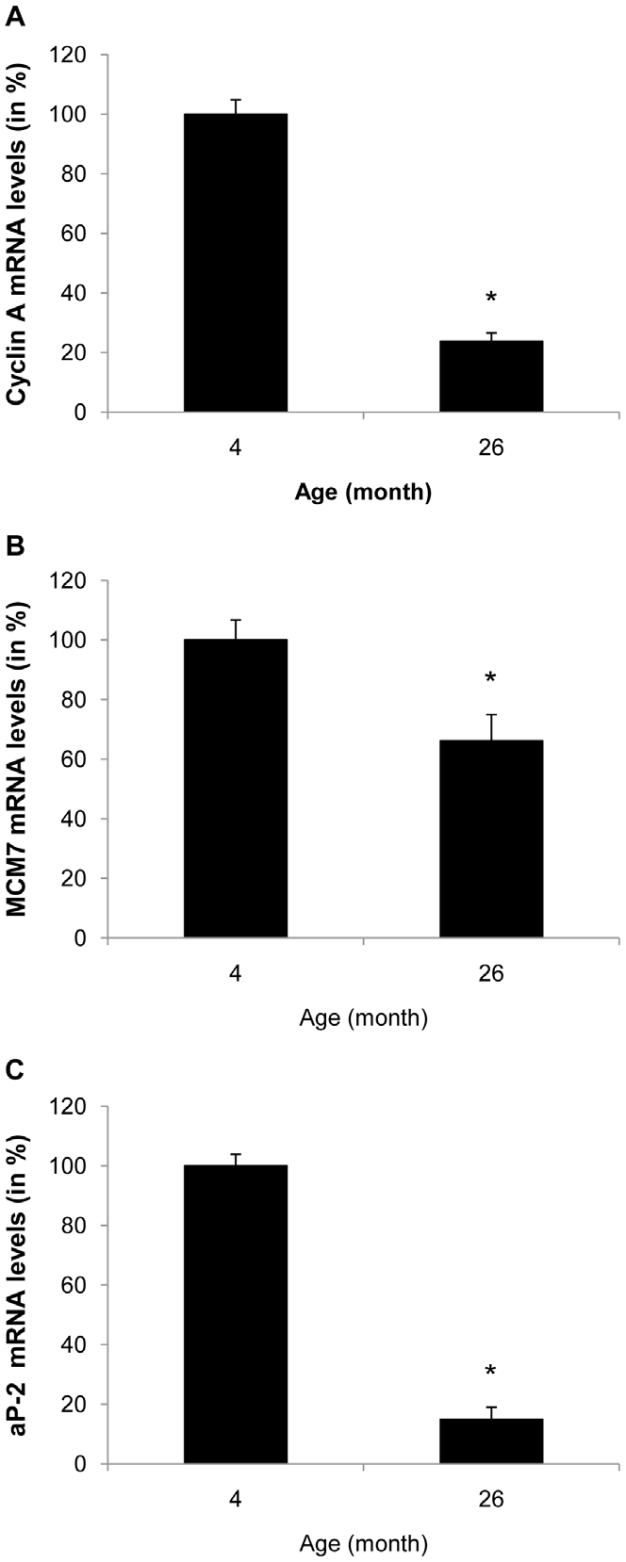
Aged adipose tissue displays reduced expression of E2F target genes and adipocyte differentiation markers. A to C: mRNA was isolated from epididymal adipose tissue of 4 and 26 month old male C57BL/6 mice. Expression of cyclin A, MCM7 and aP-2 was analyzed by real-time RT-PCR. All results are presented as mean ± SEM (* p<0.05 vs. control).

We next analyzed E2F target gene expression in adipose tissue of young and old mice that displayed an accumulation of oxidative stress during aging. As depicted in Figure 7A and B, aging was associated with a significant decline in the expression of cyclin A and MCM7 in epididymal adipose tissue. Furthermore, this decrease in E2F target gene expression was accompanied by a decrease in transcript levels of the adipocyte differentiation marker aP-2 (Figure 7C). These data demonstrate similar alterations in gene expression between aged adipose tissue and BSO treated 3T3-L1 preadipocytes.

## Discussion

Oxidative damage is postulated to be a key mechanism involved in organismal aging [24]. While oxidative stress accumulates in adipose tissue and has been implicated in insulin resistance [31], the inherent mechanisms linking oxidative damage to adipose tissue dysfunction remain unknown. In the present study, we demonstrate that aging of mice is associated with a decline in fat mass and the accumulation of oxidative stress. We further establish that oxidative stress through glutathione depletion alters adipocyte differentiation by inhibiting the mitotic clonal expansion. In concert, these data may provide a previously unrecognized mechanism contributing to age-related adipose tissue dysfunction.

Using longitudinal NMR analysis our studies documented an age-related decline of fat mass in mice, a finding that is consistent with a prior study [40]. We further demonstrate for the first time that this age-related loss of adipose tissue mass is associated with the accumulation of oxidative stress. Considering both observations and the well-established inhibition of progenitor proliferation and differentiation by oxidative damage [25], we hypothesized a causal relationship and tested whether oxidative stress alters adipogenesis. Our approach to induce oxidative stress employed the depletion of glutathione, a sophisticated non-enzymatic antioxidant defense system, allowing the accumulation of endogenously generated ROS [27]. Increased oxidative stress generated through glutathione depletion inhibited adipocyte differentiation of 3T3-L1 fibroblasts and primary stromal vascular cell fractions. Since adipocyte differentiation involves a chronologically regulated and complex network of cell cycle regulators and adipogenic transcription factors [41], we investigated the stage at which oxidative stress affects this differentiation process. Interestingly, cells treated with BSO for only the first two days of differentiation exhibited an almost similar inhibition of adipogenesis as cells treated for the entire seven days of differentiation. This first phase of differentiation is characterized by the progression of preadipocytes through one or two cell cycle divisions, referred to as mitotic clonal expansion [41]. Since glutathione depletion potently inhibited preadipocyte proliferation, these observations indicate that oxidative stress inhibits adipocyte differentiation at least in part by blocking their mitotic clonal expansion. Considering further that aging impairs the replicative potential of preadipocytes and their differentiation capacity [15], [16], [17], [18], we would infer that oxidative stress constitutes a possible mechanism underlying age-related adipose tissue dysfunction.

The mechanism by which glutathione depletion in 3T3-L1 cells inhibited mitotic expansion involved an inhibition of cell cycle progression at the G_1_→S-phase transition. Progression through the S phase is governed by the phosphorylation of Rb, which releases the repression of E2F target genes allowing transcription of S phase genes [38]. Oxidative stress exhibited no effect on the upstream CDKI or on the phosphorylation of Rb by cyclin/CDK complexes. However, the expression and transcriptional activation of the downstream E2F target genes cyclin A and MCM7 were significantly reduced in BSO-treated 3T3-L1 cells. Similarly, increased oxidative stress in adipose tissue of aged mice was accompanied by decreased expression of cyclin A and MCM7. These experiments indicate that oxidative stress alters the ability of E2F to *trans*activate its target genes, independently of Rb phosphorylation. This concept is supported by a recent study demonstrating that stress signals interfere with the E2F-dependent *trans*activation of genes required for S phase progression [42]. Although the detailed transcriptional mechanisms responsible for the silencing of E2F target genes remain unknown, it is possible that epigenetic modifications induce a transcriptionally inert chromatin environment, considering the widespread epigenetic changes occurring in response to oxidative damage [43]. Alternatively, E2F transcriptional activity may be repressed through negative cross-talk with other transcription factors. For example, oxidative stress is well established to activate inflammatory signaling, and Akari et al. recently demonstrated that NF-κB signaling represses E2F transcription factors [44]. Another group of transcription factors which may modulate E2F binding activities during adipogenesis are DP (DRTF1 polypeptide) proteins [45]. In particular, oxidative stress could potentially inhibit DP protein expression levels or its posttranslational modification, which could repress the binding activity of its heterodimeric partner E2F. Finally, Fajas et al. revealed that E2F1 deficiency in mice inhibits adipogenesis [46], lending further compelling support for a key role of E2F in the control of adipogenesis *in vivo.*

Several prior studies have established an important role of glutathione-dependent oxidative stress in adipose tissue function and metabolism *in vivo*. Consistent with our observations, a recent study by Loh et al. reported that genetic deficiency of glutathione peroxidase 1 increased reactive oxygen species and decreased fat mass expansion following high fat diet feeding [47]. Similarly, pharmacologic glutathione depletion in rats treated with BSO resulted in decreased body weight and altered insulin-stimulated glucose uptake in adipose tissue [48], [49]. However, since fat mass was not analyzed in this report, further studies on the effect of oxidative stress on energy balance and metabolism are warranted. Nevertheless, the decreased body weight in response to BSO combined with the associated adipose tissue insulin resistance noted in this study point to an important role of oxidative stress in adipose tissue biology and metabolism. This notion is further supported by studies in humans demonstrating that reduced glutathione infusion increases insulin sensitivity [50], providing further confirmation that oxidative stress alters insulin sensitivity. Considering this literature and our findings in concert, it appears conceivable to speculate that the inhibition of adipogenesis by oxidative stress may constitute an important contributor to the loss of adipose tissue mass during aging. However, further studies are warranted to confirm this hypothesis and to define the mechanisms underlying the development of insulin resistance in response to aging.
